# Inhibition of microRNA-497 ameliorates anoxia/reoxygenation injury in cardiomyocytes by suppressing cell apoptosis and enhancing autophagy

**DOI:** 10.18632/oncotarget.4774

**Published:** 2015-07-29

**Authors:** Xixian Li, Zhi Zeng, Qingman Li, Qiulin Xu, Jiahe Xie, Huixin Hao, Guangjin Luo, Wangjun Liao, Jianping Bin, Xiaobo Huang, Yulin Liao

**Affiliations:** ^1^ State Key Laboratory of Organ Failure Research, Department of Cardiology, Nanfang Hospital, Southern Medical University, Guangzhou 510515, China; ^2^ Department of Oncology, Nanfang Hospital, Southern Medical University, Guangzhou 510515, China

**Keywords:** Pathology Section, microRNA, cardiomyocytes, ischemia reperfusion, apoptosis, autophagy

## Abstract

MiR-497 is predicted to target anti-apoptosis gene Bcl2 and autophagy gene microtubule-associated protein 1 light chain 3 B (LC3B), but the functional consequence of miR-497 in response to anoxia/reoxygenation (AR) or ischemia/reperfusion (IR) remains unknown. This study was designed to investigate the influences of miR-497 on myocardial AR or IR injury. We noted that miR-497 was enriched in cardiac tissues, while its expression was dynamically changed in murine hearts subjected to myocardial infarction and in neonatal rat cardiomyocytes (NRCs) subjected to AR. Forced expression of miR-497 (miR-497 mimic) induced apoptosis in NRCs as determined by Hoechst staining and TUNEL assay. In response to AR, silencing of miR-497 using a miR-497 sponge significantly reduced cell apoptosis and enhanced autophagic flux. Furthermore, the infarct size induced by IR in adenovirus (Ad)-miR-497 sponge infected mice was significantly smaller than in mice receiving Ad-vector or vehicle treatment, while Ad-miR-497 increased infarct size. The expression of Bcl-2 and LC3B-II in NRCs or in murine heart was significantly decreased by miR-497 mimic and enhanced by miR-497 sponge. These findings demonstrate that inhibition of miR-497 holds promise for limiting myocardial IR injury.

## INTRODUCTION

Myocardial infarction (MI) is a leading cause of morbidity and mortality worldwide in patients with coronary heart disease. Early reperfusion of the ischemic region by thrombolytic treatment or percutaneous coronary intervention, although effective for salvaging the damaged myocardium, can lead to additional injury such as cardiomyocyte death and loss of cardiac function which is known as ischemia/reperfusion (IR) injury [1, 2]. The cellular mechanisms underlying IR-induced injury remain incompletely explored. MicroRNAs (miRNAs) are a class of small noncoding RNA molecules that negatively modulate gene and protein expression by promoting RNA degradation and inhibiting transcription through binding to the 3′ untranslated regions of targeted mRNAs. MicroRNAs can modulate complex physiological or disease phenotypes [3–6]. Accumulating evidence indicates the importance of several miRNAs in affecting myocardial IR injury [7–12], raising the possibility of making them potential therapeutic targets.

Recent studies have shown that the miR-15 family can worsen or alleviate myocardial ischemia and heart failure [13–15]. The miR-15 family members including miR-15a, miR-15b, miR-16, miR-195, miR-424, and miR-497, show 5′-end sequence similarity and many common targets [16, 17]. MiR-15 and miR-16 were reported to induce apoptosis by inhibiting Bcl-2 [18]. Anti-miR chemistries suppressing miR-15 in mice were reported to reduce myocardial infarct size [15], while inhibition of either miR-15a or miR-16 enhanced post-ischemic neovascularization [19]. It has been demonstrated that miR-195 is pro-apoptotic in cardiomyocytes [20]. However the exact function of miR-497 in cardiomyocytes or heart remains completely unknown.

MiR-497 is predicted to target anti-apoptosis gene Bcl-2 and autophagy gene microtubule-associated protein 1 light chain 3 B (LC3B). In non-cardiomyocytes, miR-497 has been demonstrated to promote ischemic neuronal death by downregulating Bcl-2 and Bcl-w [21], and inhibit tumorigenesis as a tumor suppressor [22, 23]. In human neuroblastoma cells, overexpression of miR-497 was shown to increase reactive oxygen species formation, disrupt mitochondrial membrane potential, and induce cytochrome C release [24]. Since both apoptosis and autophagy are deeply involved in myocardial IR injury, we designed this study to investigate the effects of miR-497 on the fate of cardiomyocytes in response to anoxia/reoxygenation (AR) or IR insult. Our data suggest that miR-497 is a new therapeutic target for myocardial IR injury.

## RESULTS

### Myocardial miR-497 expression is consistently downregulated in response to MI or AR

**Figure 1:**
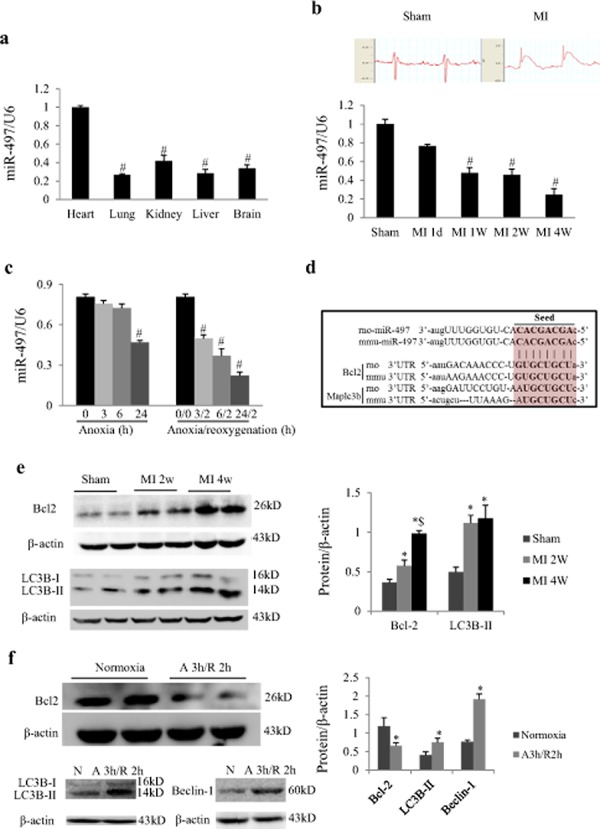
MiR-497 and its target proteins were changed in response to myocardial infarction (MI) or anoxia/reoxygenation **a.** Real-time PCR analysis indicates that the miR-497 expression was higher in adult mouse heart than in other tissues (including lung, kidney, liver and brain). ANOVA F = 60.627, ^#^*P* < 0.001 vs. Heart group. **b.** Representative electrocardiograms in mice receiving sham operation or left coronary artery ligation. Myocardial miR-497 levels were decreased in a time-dependent manner in response to MI for 1 day to 4 weeks in mice. ANOVA F = 30.053, ^#^*P* < 0.001 vs. Sham. **c.** In neonatal rat cardiomyocytes exposed to anoxia or anoxia/reoxygenation, miR497 was also changed in a time-dependent manner. Data are mean ± SEM; ANOVA F = 41.857 (anoxia treatment) and 52.590 (anoxia/reoxygenation treatment), respectively. ^#^*P* < 0.001, compared with normoxia (anoxia 0 h), for panel *A-B*, *n* = 6 per group. For panel *c*, *n* = 5. Experiments were repeated 3 times. **d.** Sequence alignment of the 3′UTR of Bcl-2 and LC3B from various species with the seed sequence of miR-497. Note the complementarity at the 5’ and 3’ ends of miR-497, where the crucial seed regions are located. **e.** Myocardial protein expressions of Bcl-2 and LC3B-II were upregulated in mice 2 or 4 weeks after MI. *P < 0.05 vs. sham, ^#x0024;^*P* < 0.05 vs. MI 2w, *n* = 6 per group. **f.** The Bcl-2, LC3B-II and beclin-1 levels in cultured neonatal rat cardiomyocytes exposed to 3 h anoxia/2 h reoxygenation (A3 h/R2 h) when compared to normoxia (N) group. *P < 0.05 vs. normoxia, *n* = 8, 5, 5 in each group for Bcl-2, LC3B-II and beclin-1, respectively. Experiments were repeated 3 times. Data are reported as mean ± SEM.

Real-time quantitative PCR showed that miR-497 was expressed in the tissues of heart, lung, kidney, liver and brain, and the expression levels were significantly higher in heart when compared with other tissues (Figure 1a). In mice with MI, myocardial miR-497 expression was dynamically down-regulated from 1 day to 4 weeks (Figure 1b). In cultured neonatal rat cardiomyocytes (NRCs) exposed to various time periods of anoxia, miR-497 expression was not changed for 3 or 6 h of anoxia but significantly decreased 24 h later (*P* < 0.001; Figure 1c). Intriguingly, miR-497 expression was dramatically decreased in a time-dependent manner when NRCs were exposed to 2 h of reoxygenation following 3–24 h of anoxia (Figure 1c). The expressions of other members of miR-15 family in cardiomyocytes or heart subjected to AR or MI or pressure overload induced by transverse aortic constriction were also investigated. We noted that miR-15 family members may be upregulated, downregulated or unchanged in response to various cardiac stresses (Supplementary Material, Figure S1). These findings indicate that miR-497 is involved in myocardial ischemia and preferentially in IR injury, suggesting that miR-497 is an IR-related microRNA in the murine heart. Based on these results, we chose 3 h anoxia/2 h reoxygenation for the following experiments.

### MiR-497 directly targets Bcl2 and LC3-B

Using bioinformatics algorithms, anti-apoptosis gene Bcl2, and autophagy gene LC3B, were predicted as putative targets of miR-497 (Figure 1d). In the heart of mice with chronic MI, we noted that both Bcl-2 and LC3B-II protein levels were increased, (Figure 1e), but in cardiomyocytes with acute AR insult, Bcl-2 protein level was decreased, while LC3B-II and beclin-1 were increased (Figure 1f).

### MiR-497 mimic induces cardiomyocyte apoptosis and inhibits autophagy

**Figure 2:**
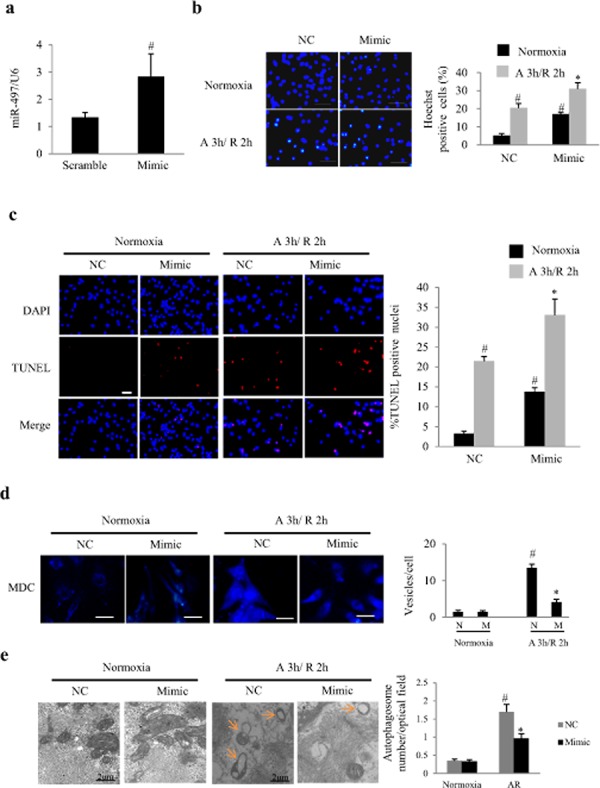
Overexpression of miR-497 enhanced apoptosis and autophagy in cultured neonatal rat cardiomyocytes (NRCs) **a.** The levels of miR-497 determined by real time-PCR in response to 24 h transfection of miR-497 mimic or negative control (NC). ^#^*P* < 0.01 vs. the NC group, *n* = 5. Experiments were repeated 3 times. **b.** Effect of miR-497 mimic on cell apoptosis determined by Hoechst staining in NRCs exposed to normoxia or anoxia/reoxygenation (A/R). Scale bar = 100 μm. **c.** Effect of miR-497 mimic on cell apoptosis determined by TUNEL in NRCs exposed to normoxia or anoxia/reoxygenation. Scale bar = 100 μm. Two-way ANOVA test was performed, F = 69.131 for treatment with miR-497 mimic or NC; F = 138.498 for treatment with normoxia or anoxia/ reoxygenation, *n* = 5. Experiments were repeated 3 times. **d.** Effect of miR-497 mimic on formation of autophagosome (indicated by arrow) which was determined by monodansylcadaverine (MDC) staining in NRCs exposed to normoxia or anoxia/reoxygenation (A/R). Scale bar = 75 μm. Each experiment was repeated 3 times. Two-way ANOVA test was performed, F = 43.090 for treatment with miR-497 mimic (M) or NC (N); F = 106.406 for treatment with normoxia or anoxia/ reoxygenation (A 3 h/R 2 h), *n* = 9 for each group. **e.** Autophagosome under electron microscopy. Scale bar = 2 μm. Two-way ANOVA F = 8.944 for treatment with miR-497 mimic or NC, *P* < 0.05, while F = 63.246 for treatment with normoxia or anoxia/ reoxygenation, *P* < 0.001. Autophagosome was counted for 100 optical fields/sample, *n* = 5 for each group. For panel *b-e*, *P < 0.05 vs. the NC group exposed to anoxia 2 h/reoxygenation 3 h (A 2 h/R 3 h), ^#^*P* < 0.05 vs. NC group exposed to normoxia.

To characterize miR-497 function, we overexpressed synthetic mature miR-497 (miR-497 mimic) in NRCs. 50 nM of miR-497 mimic was identified to increase the expression of miR-497 by more than 2 folds (Figure 2a). MiR-497 mimic significantly increased the apoptosis of NRCs even in the normoxia condition, as shown by Hoechst staining and TUNEL staining (Figure 2b and 2c). In response to AR, the apoptosis was significantly enhanced by miR-497 mimic (*P* < 0.01; Figure 2b and 2c). monodansylcadaverine (MDC) is the specific marker for autolysosomes, thus we examined the effect of miR-497 mimic on the incorporation of MDC into NRCs in response to AR. As a result, miR-497 mimics significantly inhibited autophagosome formation (Figure 2d) in cardiomyocytes during AR. Similar results were obtained using transmission electron microscopy (TEM) examination (Figure 2e). These results indicate a pro-apoptosis and an anti-autophagic role of miR-497 in cardiomyocytes.

### Silencing of miR-497 improves cell survival

**Figure 3:**
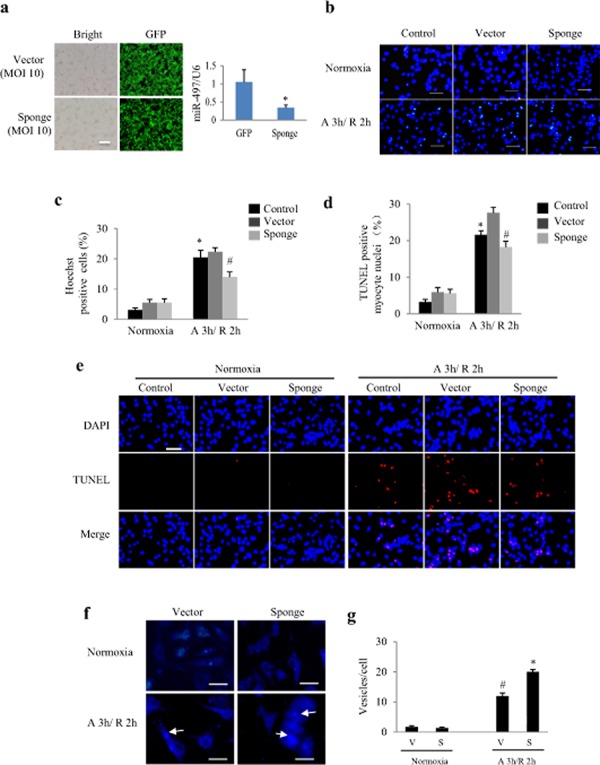
Effects of miR-497 knockdown in cultured neonatal rat cardiomyocytes (NRCs) exposed to anoxia/reoxygenation (A/R) **a.** Infective efficiency and silencing effect of adenovirus-miR-497 sponge and vector in NRCs after 24 h adenoviral infection. MOI, multiplicity of infection; GFP, green fluorescent protein, scale bar = 50 μm. *P < 0.05. **b.** Representative pictures of Hoechst staining, Scale bar = 100 μm. **c.** Hoechst staining shows that miR-497 sponge-treated cardiomyocytes were resistant to A/R-induced apoptosis. Two-way ANOVA analysis was performed, F = 3.456 for treatment with miR-497 sponge or vector or control, *P* < 0.05; F = 131.769 for treatment with normoxia or anoxia/ reoxygenation (A 3 h/R 2 h), *P* < 0.001. *P < 0.001 vs. control group under normoxia, ^#^*P* < 0.05 vs. vector group under A/R. **d.** TUNEL assay shows that miR-497 sponge-treated cardiomyocytes were resistant to A/R-induced apoptosis. Scale bar = 100 μm. Two-way ANOVA analysis was performed, F = 6.182 for treatment with miR-497 sponge or vector or control, *P* < 0.01; F = 199.523 for treatment with normoxia or A 3 h/R 2 h, *P* < 0.001. *P < 0.001 vs. control group under normoxia, ^#^*P* < 0.05 vs. vector group under A/R. **e.** Representative pictures of TUNEL staining. **f.** Autophagy detected by monodansylcadaverine (MDC) staining (the arrows indicate the autophagosomes). Scale bar = 75 μm. **g.** Quantification of vesicle number in each cardiomyocyte. Two-way ANOVA analysis was performed, F = 29.254 for treatment with miR-497 sponge (S) or vector (V), *P* < 0.001; F = 403.582 for treatment with normoxia or A 3 h/R 2 h, *P* < 0.0001. *P < 0.001 vs. control group under normoxia, #*P* < 0.001 vs. vector group under A/R. Data are mean ± SEM, n = 5 for each group, and each experiment was repeated 3 times.

To further examine the contribution of miR-497 to AR injury, NRCs were transfected with the Ad-miR-497-sponge. As shown in Figure 3a, a satisfactory infection efficiency of miR-497 sponge and silencing effect were obtained. Cell apoptosis was determined by Hoechst and TUNEL staining (Figure 3b-e). The Hoechst and TUNEL staining analyses showed that miR-497-sponge exerted no influence on cardiomyocytes under normoxia, but significantly decreased apoptosis in cardiomyocytes with AR insult (*P* < 0.05, Figure 3c and 3d). Autophagy detected by MDC was significantly enhanced in miR-497 sponge-treated NRCs than in vector-treated cells (Figure 3f and 3g). These results indicate that silencing of miR-497 attenuates AR injury in cardiomyocytes.

### Effects of miR-497 expression on protein expression related to autophagy and apoptosis

**Figure 4:**
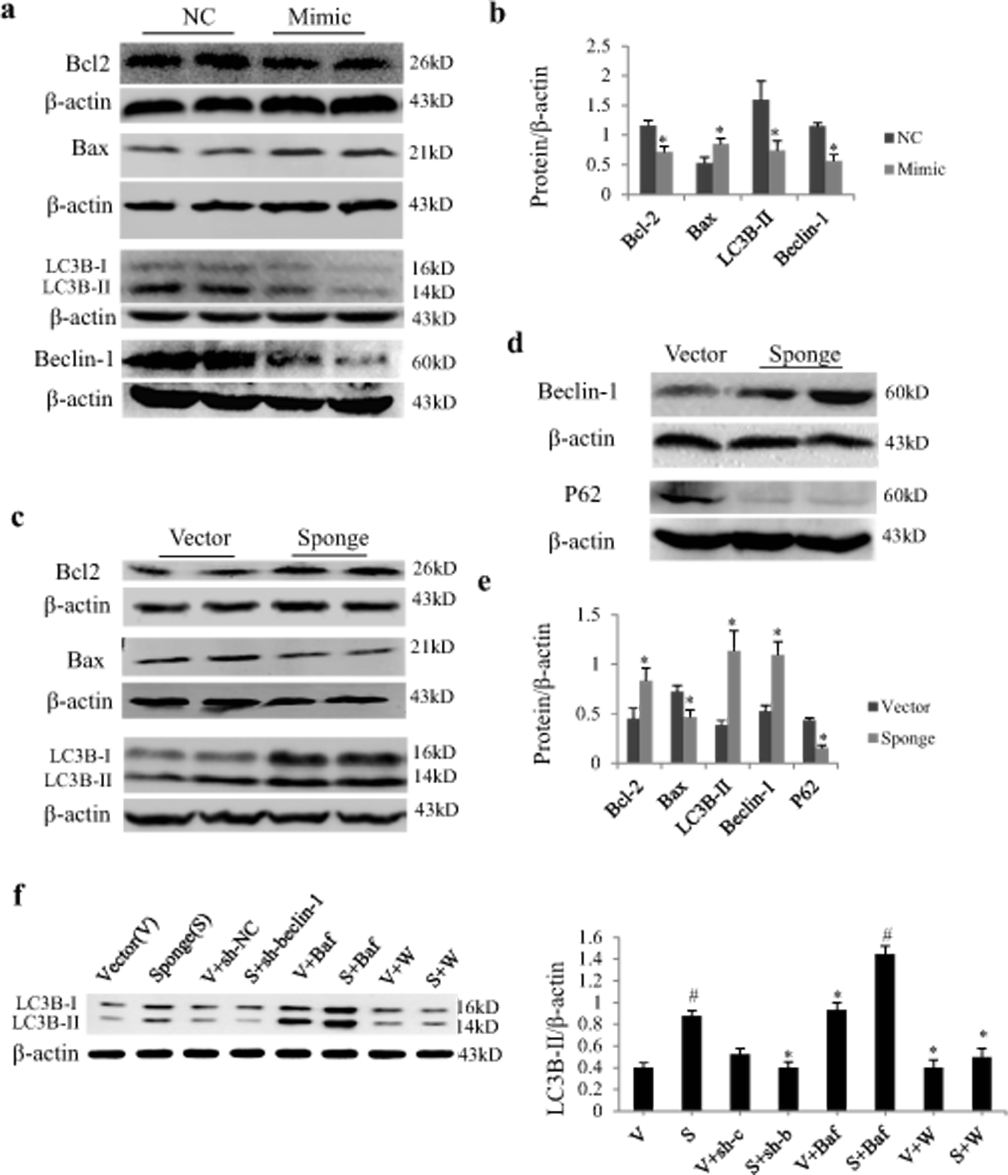
Effect of miR-497 mimic and sponge on the expression of Bcl2 and LC3B protein in neonatal rat cardiomyocytes (NRCs) **a.** NRCs were transfected with miR-497 mimic or negative control (NC). Western blot shows the effects of miR-497 overexpression on Bcl-2, Bax, LC3B-II and beclin-1. **b.** Quantitation for panel A. NRCs were transfected with miR-497 sponge or vector adenovirus. Western blot shows the protein expression of Bcl-2, Bax and LC3B-II **c.** beclin-1 and P62 **d.** *which* were significantly changed in miR-497 sponge-treated cells **e, f.** Western blotting of LC3B-II in NRCs treated with/without miR-497 sponge (S), vector (V), Ad-sh-beclin-1 (sh-b), Ad-sh-control (sh-c), bafilomycin A1 (Baf), wortmannin (W). Data are mean ± SEM, *n* = 5–7 for each group; for panel b and e, *P < 0.05, compared with the corresponding NC or sponge group, while for panel f, *P < 0.05 vs. sponge group, ^#^*P* < 0.05 vs. vector group.

In cultured NRCs, Western blot analysis showed that miR-497 mimic significantly down-regulated protein expressions of Bcl2, LC3B-II and beclin-1, and up-regulated Bax expression (Figure 4a and 4b). MiR-497 sponge exerted opposite effects and increased autophagic influx as determined by P62 downregulation (Figure 4c–4e). By using adenovirus carrying short hairpin RNA for becelin-1 (sh-beclin-1) and lysosomal inhibitor Bafilomycin A1 (Baf), we further investigated whether the increase of LC3B-II by miR-497 sponge is affected by beclin 1. As shown in Figure 4f, the upregulation of LC3B-II was enhanced by Baf and antagonized by Ad-sh-beclin-1 and autophagic inhibitor wortmannin.

### MiR-497 regulates autophagy and apoptosis related signal proteins in the context of AR

**Figure 5:**
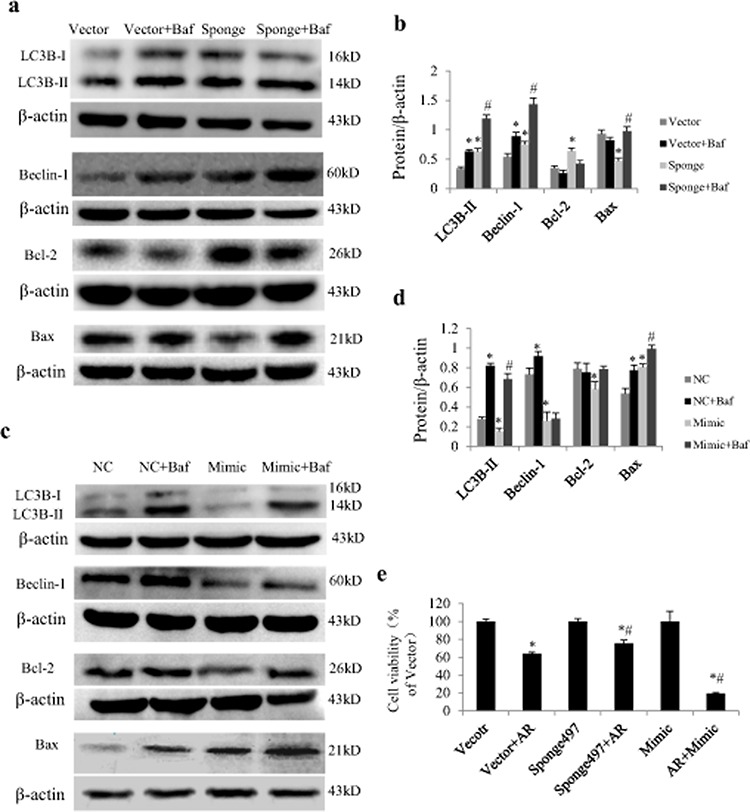
Effect of miR-497 on proteins related to autophagy and apoptosis in neonatal rat cardiomyocytes (NRCs) exposed to anoxia/reoxygenation (A/R) **a.** Examples of western blotting showing effects of miR-497 sponge and Baf on LC3B-II, beclin-1, Bcl-2 and Bax. **b.** Quantitation of panel A. **c.** Examples of western blotting showing effects of miR-497 mimic and Baf on LC3B-II, beclin-1, Bcl-2 and Bax. **d.** Quantitation of panel C. **e.** Results of MTT cell viability assay showing effect of miR-497 on cell viability in the presence of A 3 h/R 2 h. Data are mean ± SEM, *n* = 5 for each group. For panel b and d, *P < 0.05 vs. Vector or NC (negative control) group, ^#^*P* < 0.05 vs. Sponge or Mimic group. For panel e, *P < 0.05 vs. vector group, ^#^*P* < 0.05 vs. vector + AR group. Sponge indicates miR-497 sponge, Mimic indicates miR-497 mimic.

In cultured NRCs exposed to AR, loss and gain function of miR-497 also exerted significant influence on protein expression of LC3B-II, beclin-1, Bcl-2 and Bax. We noted that miR-497 mimics decreased Bcl-2, LC3B-II and beclin-1 and increased Bax in cardiomyocytes in the presence of AR (Figure 5a–5b), while opposite effects were noted by treatment with miR-497 sponge (Figure 5c–5d). Inhibiting lysosomal degradation with Baf increased beclin-1 and LC3B-II expression levels in the presence of miR-497 mimic or sponge (Figure 5a–5d). Addition of Baf to miR-497 mimic or sponge increased Bax expression, suggesting that inhibition of autophagic flux promotes apoptosis (Figure 5a–5d). MTT (3-(4,5-dimethyl thiazol-2-yl)-2,5-diphenyl tetrazolium bromide) cell viability assay shows that inhibition of miR-497 increased cell survival in the context of AR (Figure 5e).

### MiR-497 sponge reduces myocardial infarct size in mice

**Figure 6:**
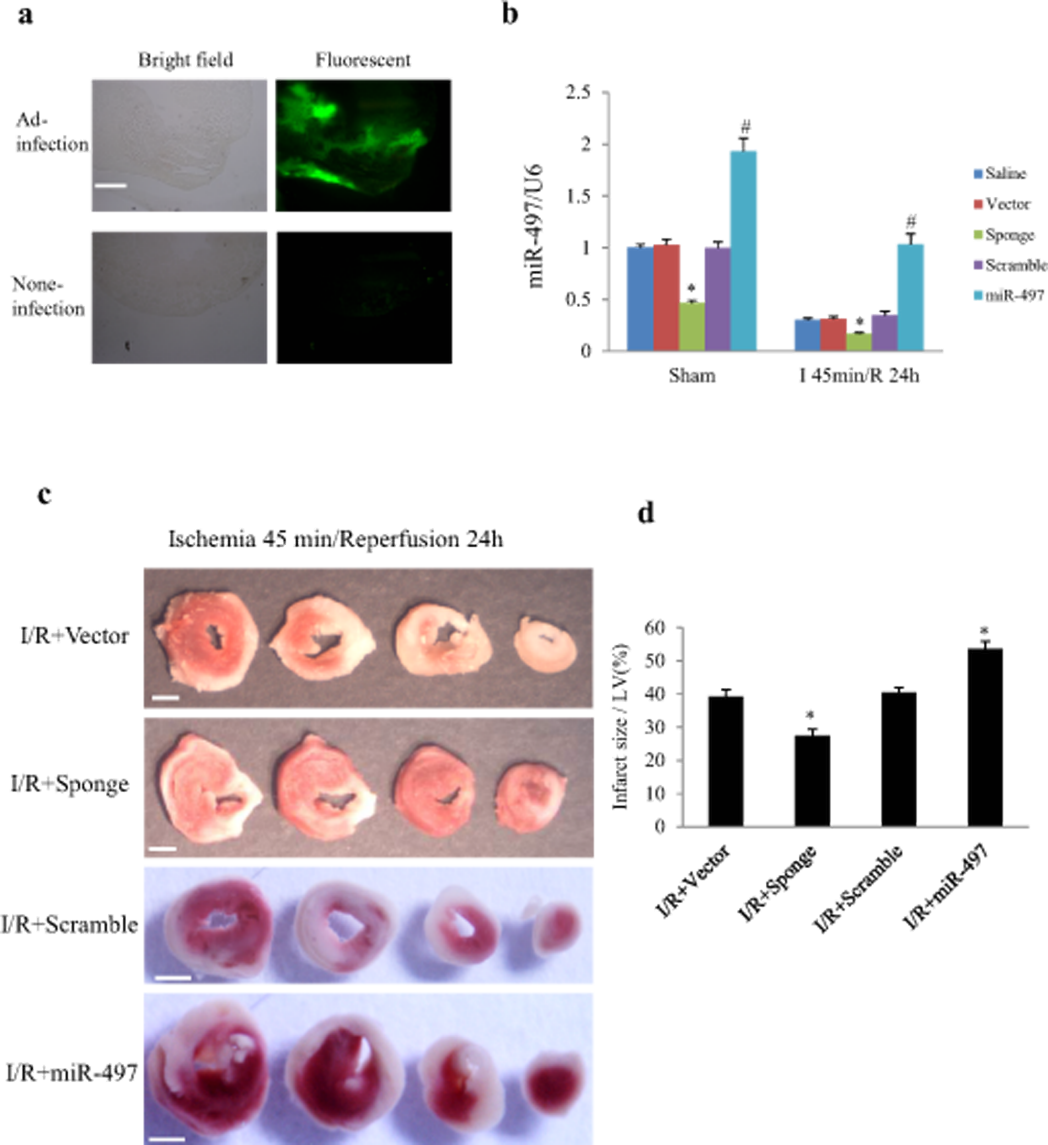
MiR-497 exerted influence on myocardial infarct size in response to myocardial ischemia/reperfusion (I/R) C57 BL/6 mice were subjected to myocardial ischemia for 45 min followed by reperfusion for 24 h or sham operation. Adenovirus carrying miR-497-sponge or miR-497 were directly injected into the myocardium. **a.** Transduction efficiency of adenovirus was examined in cryosectioned mouse heart. Three days after intra-myocardial injection, more than 50% of the transduction efficiency was reached (judged from green fluorescence emitted by EGFP from adenovirus). Scale bar = 500 μm. **b.** Levels of myocardial miR-497 expression determined by real-time-PCR in different groups. Two-way ANOVA analysis was performed, F = 92.271 for treatment with adenovirus or saline; F = 259.673 for treatment with sham or I/R. *P < 0.001 vs. corresponding vector group, ^#^*P* < 0.001 vs. the corresponding scramble group, *n* = 5 in each group. **c.** Representative pictures of TTC staining in 4 hearts from different IR groups (white color indicates infarct myocardium, red color indicates viable tissue). Scale bar = 1 mm. **d.** Quantitation of myocardial infarct size in 4 IR groups treated with scramble, miR-497 sponge, vector and miR-497 mimic, respectively. ANOVA F = 31.255, *P < 0.001, *n* = 6 per group. Data are mean ± SEM. Sponge, adenovirus-miR-497 sponge; miR-497, adenovirus-miR-497.

**Figure 7:**
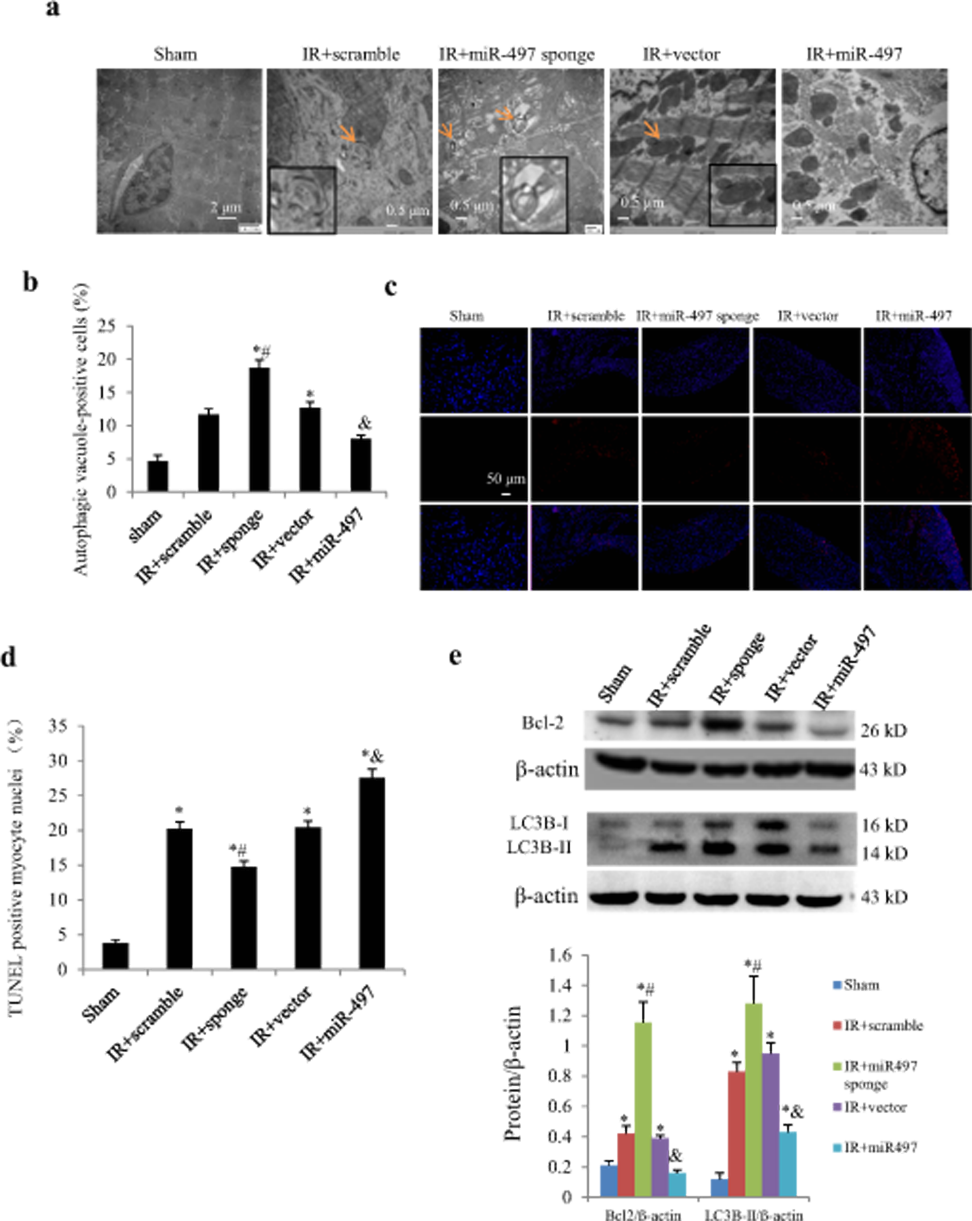
Effects of Ad-miR-497 sponge and Ad-miR-497 on myocardial autophagy and apoptosis in mice subjected to ischemia/reperfusion (IR) **a.** Autophagic vacuole in response to different treatments examined by transmission electron microscopy. Arrows indicate autophagosome (double membrane structure, also see the inserts). **b.** Quantitative analysis of the number of autophagic vacuole-positive cells (*n* = 5 in each group). Data are reported as mean ± SEM. *P < 0.01 vs. Sham, ^#^*P* < 0.01 vs. IR+scramble, ^&^*P* < 0.05 vs. IR+vector. **c.** Representative pictures of TUNEL staining. **d.** The quantitative TUNEL assay (*n* = 5 in each group). **e.** Western blotting of Bcl2 and LC3B (*n* = 5 in each group). *P < 0.05 vs. Sham, ^#^*P* < 0.05 vs. IR+scramble, ^&^*P* < 0.05 vs. IR+vector. Sponge = adenovirus-miR-497 sponge; miR-497 = adenovirus-miR-497.

To elucidate the *in vivo* effects of miR-497 inhibition on IR injury, we directly injected Ad-miR-497-sponge or Ad-miR-497 into left ventricular myocardium of mice 3 days before the onset of IR procedure. As shown in Figure 6a, 3 days after intra-myocardial injection, more than 50% adenovirus transduction efficiency was reached. Consistently, miR-497 levels were significantly decreased when the mice were subjected to ischemia for 45 min and reperfusion for 24 h. Meanwhile, miR-497 levels were further reduced in Ad-miR-497-sponge-treated group but significantly increased in Ad-miR-497-treated group (Figure 6b). Myocardial infarct size determined with triphenyltetrazolium chloride (TTC) staining was significantly smaller in mice treated with Ad-miR-497-sponge than in mice receiving scramble vector infection, while myocardial infarct size was larger in Ad-miR-497-treated mice than in control vector-treated group (Figure 6c and 6d). Similar to the results in cultured NRCs, treatment with Ad-miR-497-sponge enhanced cardiomyocyte autophagy and inhibited apoptosis in IR mice, while Ad-miR-497 exerted opposite effects (Figure 7a–7e).

## DISCUSSION

In the present study, we identified miR-497 as a critical regulator of cardiomyocyte apoptosis and autophagy via targeting Bcl-2 and LC3B genes. We found that miR-497 was enriched in cardiac tissue and was dramatically downregulated in response to IR or AR insult. Furthermore, overexpression of miR-497 increased cardiomyocyte apoptosis and inhibited autophagic flux, whereas silencing of miR-497 reduced cardiomyocyte apoptosis and enhanced autophagic flux. The cardioprotective effect of anti-miR-497 was further confirmed in mice with IR insult.

The regulation of miR-15 family is spatial, temporal and dynamic [15, 17]. Their expression profile depends on species and type of pathological stress. It was reported that miR-15 family members were upregulated in infarct region of pigs [15] but downregulated in both border and infarct zone of mice [17] in response to myocardial infarction, while they were found to be up-regulated in the overloaded heart in multiple species [19]. The expression heterogeneity of miR-15 family members was also supported by previous studies. Roy et al reported that only miR-15b but no other family members was upregulated in mouse heart subjected to IR for 2 or 7 days [25], while in rats with cerebral IR for 24 h or 48 h, only miR-497 in brain tissue was significantly upregulated [26]. Although miR-15 family members share similar structure and some common targets, they can also exert distinct role in the pathogenesis of cardiovascular disease. It was reported miR-195 increases cardiac hypertrophy [27] and worsens systolic dysfunction in mice with MI [17], while miR-15b, another member of the same miR-15 family, was found to attenuate myocardial fibrosis and hypertrophy in pressure-overloaded mice [19]. Therefore, it is of significance to delineate the respective roles of family members.

Cardiomyocyte apoptosis is a well-known key cellular event in ischemic hearts [28]. Increasing evidence indicates the importance of miRNAs in the regulation of various cardiovascular diseases including myocardial IR injury. Christine et al examined the expression of 257 microRNAs in peripheral blood mononuclear cells derived from patients with chronic heart failure, and found that miR-497was significantly decreased in patients with ischemic cardiomyopathy [29], which is in consistence with our findings in this study. Accumulating evidence shows that miR-497 is involved in the regulation of apoptosis. In mouse N2A cells after oxygen-glucose deprivation, miR-497 was found to promote apoptosis by targeting Bcl-2 and Bcl-w [21]. In both gastric and lung cancer cell lines, miR-497 modulates apoptosis by targeting Bcl2 [30]. In the heart, Bcl2 is involved in myocyte cell loss and contributes to a variety of cardiac pathologies, including heart failure and IR injury [31, 32]. Overexpression of Bcl-2 in mice attenuates apoptosis and alleviates myocardial IR injury [33]. Similar to our findings on miR-497, Hullinger et al has demonstrated that miR-15b, also a member of miR-15 family, aggravates myocardial IR injury by targeting Bcl-2 [15]. In consistent with previous reports [21, 34], we noted that Bcl-2 protein was increased in murine heart with MI for several weeks, but it was decreased in cultured cardiomyocytes in response to acute AR. A possible explanation for this discrepancy is that a compensatory increase of myocardial Bcl-2 may occur in mice with chronic MI but not in cultured cardiomyocytes with acute AR. Although the downregulation of miR-497 in response to AR would expect an increase of Bcl-2 in cardiomyocytes, other proapoptotic factors may antagonize this effect and eventually lead to a net effect of Bcl-2 downregulation. In this study, we clearly demonstrated that overexpression or knockdown of miR-497 alone decreased and increased Bcl-2 protein, respectively. Although Bax is not a predicted target gene of miR-497, its expression can be regulated by miR-497, which is likely secondary to the change of Bcl-2, a similar phenomenon was also reported elsewhere [12]. Bcl-2 can operate as upstream regulator that opposes the intrinsic death-inducing actions of Bax [35].

In addition, autophagy is commonly observed in the heart with acute and chronic ischemia and heart failure [36, 37]. There is substantial evidence that autophagy is up-regulated in ischemic-reperfused cardiomyocytes [38]. Whether autophagy induced during reperfusion is beneficial or detrimental remains controversial. Hamacher-Brady et al reported that in the cardiac HL-1 cells, I/R-induced autophagy and apoptosis were decreased strikingly when autophagy was increased, which suggested it plays a cardioprotective role in response to cardiac IR injury [38]. In contrast, Matsui et al showed that excessive autophagy with robust upregulation of BECN1 during reperfusion appeared to enhance cell death, which was detrimental to the heart [39]. There is general agreement that it is a homeostatic mechanism, thus insufficient or excessive autophagy is detrimental to maintain cardiac function. In this study, we found downregulated miR-497 could moderately increase autophagy and reduce myocardial damage during IR (45 min/24 h) and AR (3 h/2 h), which indicate that a moderate enhancement of autophagy would attenuate myocardial injury induced by IR. In cultured cardiomyocytes under normoxia, change of miR-497 expression levels exerted influence on autophagic signal but did not lead to detectable change of vesicle formation as measured by non-selective MDC staining or EM, while under AR, miR-497 significantly regulated both autophagic flux and vesicle formation.

The downregulation of miR-497 in response to myocardial ischemia or IR may work as an important adaptive mechanism to upregulate the expression levels of Bcl2 and LC3B. Silencing of endogenous miR-497 provides protection against IR-induced cardiomyocyte death and apoptosis by targeting Bcl-2 and LC3B. Thus, miR-497 and its downstream targets may serve as valuable therapeutic entry points and thereby improve cardiac performance after IR injury. Because a single microRNA can regulate a number of different mRNAs, and the same mRNA can be silenced by multiple miRs, it is therefore important to further explore the target network of miR-497 in ischemic diseases. Because complementary sites on the lncRNA (long noncoding RNA) allow them to recognize and bind to miRNAs and act as highly specific sensors for their regulation, identifying the lncRNA that regulates miR-497 would also be helpful for searching new therapeutic targets of myocardial IR.

## MATERIALS AND METHODS

### Agents

3-(4,5-dimethyl thiazol-2-yl)-2,5-diphenyl tetrazolium bromide (MTT), bafilomycin A1 (Baf), wortmannin, monodansylcadaverine (MDC), 3-(4,5-dimethyl thiazol-2-yl)-2,5-diphenyl tetrazolium bromide (MTT), triphenyltetrazolium chloride (TTC) were purchased from Sigma. Antibodies against beclin-1 (3495), LC3B (#2775), p62(5114S) and Bax were obtained from Cell Signaling Technology, Danvers, Massachusetts, USA, anti-Bcl-2(SC-7382) was from Santa Cruz Biotechnology, USA. β-actin (TA-09) was from ZSGB-BIO, Beijing, China. Antimyoactin antibody, was obtained from Wuhan Boster Biological Technology, LTD., China. Lipofectamine RNAiMAX kit was purchased from Invitrogen Company.

### Mouse MI and IR model

Mice (C57BL/6, male, weighed 20–25 g, aged 8–10 weeks) were subjected to intraperitoneal anesthesia with a mixture of xylazine (5 mg/kg) and ketamine (100 mg/kg). The left coronary artery (LCA) permanent ligation or ligation for 45 min followed by 24 h of reperfusion was carried out as we described previously [40]. Ischemia was confirmed by myocardial blanching and continuous electrocardiography monitoring showing ST segment elevation. The IR mice were sacrificed by overdose anesthesia (pentobarbital sodium 150 mg/kg intraperitoneal) and cervical dislocation 24 h after surgery for the measurement of infarct size with TTC staining.

All procedures were performed in accordance with our Institutional Guidelines for Animal Research and the Guide for the Care and Use of Laboratory Animals published by the National Institutes of Health (NIH Publication, 8th Edition, 2011).

### Cell culture and transfection

The neonatal rats were sacrificed by anesthesia with 2% isoflurane inhalation and their hearts were then removed. Neonatal rat cardiomyocytes (NRCs) were isolated from 1-to 2-day-old Sprague-Dawley rats. Cells isolated from 3 hearts were used as one sample. The confirmation of cell type was performed by using immunochemistry assay (antimyoactin antibody) as described in our previous study [18]. The planting density of cardiomyocytes was 1.2–1.5 × 10^5^/cm^2^ for RNA and protein extraction, 0.6–0.7×10^5^/cm^2^ for morphological observation. Cells were incubated in Dulbecco's Modified Eagle Medium (DMEM, Gibco, Invitrogen, Carlsbad, CA, USA) with 10% fetal bovine serum (Gibco). Either specific mimic or anti-RNA was transfected to overexpress or to silence miR-497 in the cultured cells. Transfections of miR-497 mimic were performed with a Lipofectamine RNAiMAX kit (Invitrogen) according to the manufacturer's instructions. Double-stranded miR-497 mimic and the miRNA negative control (NC) (GenePharma, Shanghai, China) at a final concentration of 50 nM were introduced into the NRCs. Transfected cells were harvested 24 h later.

### Anoxia/reoxygenation in NRCs and cell viability assay

Immediately after transfection of miR-497 mimic or sponge or vector, NRCs were placed in either a low oxygen atmosphere or normoxia conditions. Normoxia condition was created in a normoxia incubator with 21% O_2_ and 5% CO_2_ at 37°C. To induce anoxia, NRCs were incubated in a humidified environment at 37°C in a 3-gas hypoxic chamber maintaining 5% CO_2_ and 1% O_2_ (oxygen was expelled by nitrogen) for various time periods (3–24 h). After anoxia, NRCs were exposed for 2 h of reoxygenation under normoxia condition. The cells that were not subjected to AR were incubated under normoxia for the same duration and served as control. The cells were harvested for the isolation of cellular RNA and protein. Cell viability of the cultured cardiomyocytes in response to various treatments was determined using MTT assay according to the manufacture's instruction.

### Real-time PCR assay of microRNAs

Total RNA was extracted from NRCs or heart tissues using E.Z.N.A™ Total RNA Kit II (OMEGA, Norcross, GA, USA) in accordance with the manufacturer's protocol. The concentration of RNA was measured with a NanoDrop Spectrophotometer (NanoDrop Tech, Rockland, Del). For reverse transcription and quantitative real-time polymerase chain reaction (PCR), 10 ng of total RNA per sample was used with the All-in-One TM miRNA Q-PCR Detection Kit (Fulengen, Rockville, MD, USA). Specific primers for miR-15 family members were obtained from GeneCopoeia Inc. Quantitative real-time PCR was conducted using a Quantitect SYBR Green RT-PCR kit (TaKaRa Bio, Tokyo, Japan) and an Applied Biosystems 7500 system. MicroRNA-497 levels were quantified with the 2^(−ΔΔct)^ relative quantification method that was normalized to the snRNA U6.

### Western blot analysis

Protein extraction was performed in lysis buffer containing protease inhibitors (Sigma, St. Louis, MO, USA) and Western blotting was performed following the standard procedures. Protein lysates were electrophoresed on 15% SDS-PAGE gels, and transferred onto nitrocellulose membranes (iBLOT; Invitrogen). The membranes were incubated with primary antibodies against Bcl2, LC3B, p62, Bax, beclin-1. Blotting of β-actin (ZSGB-BIO, Beijing, China) was used as a loading control. The signals were quantified using the Image J software (National Institute of Health, Bethesda, MD). The target protein expression levels were normalized towards the corresponding loading control.

### Apoptosis detection and MDC labeling

NRCs were incubated in 35-mm Petri dishes. Apoptosis was analyzed by terminal deoxynucleotidyl transferase dUTP nickend labeling (TUNEL, Roche, USA) and Hoechst 33258 (Beyotime, Shanghai, China) according to the manufacturer's instructions.

Autophagosomes in NRCs were labeled with MDC staining as described elsewhere [41]. After incubation with 50 nmol/L MDC in phosphate buffered saline (PBS) at 37°C for 10 min, the NRCs were washed with PBS and fixed with 4% paraformaldehyde.

The apoptosis nucleus and autophagic vacuoles were visualized with confocal microscope.

### Overexpression and silencing of miR-497 and becline-1 knockdown

#### Preparation of MiR-497 mimic and negative control (NC)

They were synthesized by GenePharma Company with the following sequences: rno-miR-497 mimics (5′-cagcagcacacugugguuugua-3′) and miRNA NC (5′-uucuccgaacgugucacgutt-3′) which shares minimal sequence identity in mammals.

#### Preparation of adnovirus-miR-497 sponge

To inhibit the activity of miR-497, a U6 sponge was used. Replication deficient adenoviruses for miR-497 sponge and null virus were prepared using the Getaway System (Invitrogen) according to the manufacturing's instructions. Briefly, the oligonucleotides for miR-497 were synthesized by Shanghai General Biotech with the following sequences: 5′-gtacaaaccacagtgtgctgctgcgtatacaaaccacagtgtgctgctggcatg-3′ (forward) and 5′-ccagcagcacactgtggtttgtatacgcagcagcacactgtggtttgtactgca-3′ (reverse), which contained two perfect miR-497 binding sites. Then the double-strand oligonucleotide was cloned into a shpDown-U6 RNA-eGFP plasmid (Cyagen, Beijing, China) and confirmed by sequencing. MiR-497 oligo/shpDwon-U6 RNA-eGFP plasmid was recombined with pAD/PL-DEST (Invitrogen) using LR recombinase (Invitrogen) which was confirmed by sequencing. The pEntr-U6-miR-497-sponge (Ad-miR-497-spong) was transfected to 293A cells using lipofectamine 2000. Control vector was prepared by transfecting 293A with pEntr-U6-scrambled oligonucleotide-eGFP (control vector). Viruses were purified using the Adenovirus purification mini Kit (V1160-01, Biomiga, San Diego, USA). The end-point dilution assay was used to measure virus titer.

#### Preparation of adenovirus-miR-497

Construction of plasmid pDC316-mCMV-EGFP-CMV-mir-497 and adenovirus packaging of the recombinant plasmid were completed by a professional company (Biowit Technologies, Shenzhen, China). Mouse miR-497 (synthetized by Invitrogen) was inserted into vector pDC316-mCMV-EGFP by using restriction enzymes NheI and HindIII. The mir-497 clones were sequenced completely to confirm the absence of cloning artifacts and mutation. The AdMAXTM system was used for the generation of recombinant adenovirus carrying miR-497 or empty vector (pEGFP). Briefly, pDC316-mCMV-EGFP-miR-497 and virus backbone plasmid pBHGloxdelIE13cre were co-transfected into cultured HEK293 cells by using polyfectamine, then the recombinant adenovirus were collected and amplified in HEK293 cells.

#### Preparation of adenovirus-short hairpin RNA of beclin-1(Ad-sh-beclin-1)

Ad-sh-beclin-1 and Ad-scramble were generated by a professional company (Vigene Biosciences, Shandong, China) as we reported elsewhere [42]. The infection efficiency was satisfactory as confirmed with fluorescence microscopy and western blotting (Supplementary Material, Figure S2).

#### Infection approaches

The Ad-miR-497-sponge or miR-497 mimic or Ad-sh-beclin-1 was directly transfected to the cultured NRCs (multiplicity of infection (MOI) = 10), while *in vivo* infection of Ad-miR-497-sponge or Ad-miR-497 in heart was reached by direct myocardial injection through a left thoracotomy in mice anesthetized with a mixture of xylazine (5 mg/kg, intraperitoneal) and ketamine (100 mg/kg, intraperitoneal) as reported elsewhere [35]. Briefly, Ad-miR-497-sponge or Ad-miR-497 or the corresponding control virus particles (1 × 1011 viral genomes/ml) were administered by direct injection in the left ventricular free wall (3 sites, 20 μl/site) using a syringe with a 30-gauge needle, and 3 days later, sham or IR surgery was performed. Transduction efficiency of *in vivo* gene transfer by adenovirus was assessed by enhanced green fluorescent protein (EGFP) fluorescence (510 nm) in cryosectioned heart slices using a fluorescence microscopy.

### Electron microscopic examination

Cultured NRCs or murine heart sections were prepared for transmission electron microscopy (TEM) to investigate the formation of autophagosome. For cardiac tissue preparation, mice under anesthesia were perfused from the left ventricle of the heart with normal saline and then with 2% paraformaldehyde plus 2% glutaraldehyde in 0.1 M phosphate buffer (pH 7.4). The heart was removed and cut into 1 mm^3^ blocks that were then fixed in 2.5% glutaraldehyde overnight at 4°C, and post-fixed in 1% buffered osmium tetroxide. The specimens were conventionally processed and examined under a TEM (H-800; Hitachi).

### Statistical analysis

Quantitative data were expressed as mean ± SEM (standard error of mean). Comparison between two experimental groups was based on a two-tailed *t*-test, while comparisons of parameters among ≥3 groups were analyzed by one-way or two-way ANOVA followed by Bonferroni's correction for post hoc multiple comparisons. In all analyses, differences were considered statistically significant at a value of *P* < 0.05.

## SUPPLEMENTARY FIGURES



## References

[1] Dirksen MT, Laarman GJ, Simoons ML, Duncker DJ (2007). Reperfusion injury in humans: a review of clinical trials on reperfusion injury inhibitory strategies. Cardiovasc Res.

[2] Frohlich GM, Meier P, White SK, Yellon DM, Hausenloy DJ (2013). Myocardial reperfusion injury: looking beyond primary PCI. Eur Heart J.

[3] Zhao Y, Samal E, Srivastava D (2005). Serum response factor regulates a muscle-specific microRNA that targets Hand2 during cardiogenesis. Nature.

[4] Chen JF, Mandel EM, Thomson JM, Wu Q, Callis TE, Hammond SM, Conlon FL, Wang DZ (2006). The role of microRNA-1 and microRNA-133 in skeletal muscle proliferation and differentiation. Nature genetics.

[5] Care A, Catalucci D, Felicetti F, Bonci D, Addario A, Gallo P, Bang ML, Segnalini P, Gu Y, Dalton ND, Elia L, Latronico MV, Hoydal M, Autore C, Russo MA, Dorn GW (2007). MicroRNA-3 controls cardiac hypertrophy. Nat Med.

[6] Mishra PK, Tyagi N, Kumar M, Tyagi SC (2009). MicroRNAs as a therapeutic target for cardiovascular diseases. Journal of cellular and molecular medicine.

[7] Thum T, Gross C, Fiedler J, Fischer T, Kissler S, Bussen M, Galuppo P, Just S, Rottbauer W, Frantz S, Castoldi M, Soutschek J, Koteliansky V, Rosenwald A, Basson MA, Licht JD (2008). MicroRNA-21 contributes to myocardial disease by stimulating MAP kinase signalling in fibroblasts. Nature.

[8] Wang X, Zhang X, Ren XP, Chen J, Liu H, Yang J, Medvedovic M, Hu Z, Fan GC (2010). MicroRNA-494 targeting both proapoptotic and antiapoptotic proteins protects against ischemia/reperfusion-induced cardiac injury. Circulation.

[9] Ye Y, Hu Z, Lin Y, Zhang C, Perez-Polo JR (2010). Downregulation of microRNA-29 by antisense inhibitors and a PPAR-gamma agonist protects against myocardial ischaemia-reperfusion injury. Cardiovasc Res.

[10] Fiedler J, Jazbutyte V, Kirchmaier BC, Gupta SK, Lorenzen J, Hartmann D, Galuppo P, Kneitz S, Pena JT, Sohn-Lee C, Loyer X, Soutschek J, Brand T, Tuschl T, Heineke J, Martin U (2011). MicroRNA-24 regulates vascularity after myocardial infarction. Circulation.

[11] Wang X, Ha T, Liu L, Zou J, Zhang X, Kalbfleisch J, Gao X, Williams D, Li C (2013). Increased expression of microRNA-146a decreases myocardial ischaemia/reperfusion injury. Cardiovasc Res.

[12] Ahmad F, Lal H, Zhou J, Vagnozzi RJ, Yu JE, Shang X, Woodgett JR, Gao E, Force T (2014). Cardiomyocyte-specific deletion of Gsk3alpha mitigates post-myocardial infarction remodeling, contractile dysfunction, and heart failure. Journal of the American College of Cardiology.

[13] Thum T, Galuppo P, Wolf C, Fiedler J, Kneitz S, van Laake LW, Doevendans PA, Mummery CL, Borlak J, Haverich A, Gross C, Engelhardt S, Ertl G, Bauersachs J (2007). MicroRNAs in the human heart: a clue to fetal gene reprogramming in heart failure. Circulation.

[14] van Rooij E, Sutherland LB, Thatcher JE, DiMaio JM, Naseem RH, Marshall WS, Hill JA, Olson EN (2008). Dysregulation of microRNAs after myocardial infarction reveals a role of miR-29 in cardiac fibrosis. Proceedings of the National Academy of Sciences of the United States of America.

[15] Hullinger TG, Montgomery RL, Seto AG, Dickinson BA, Semus HM, Lynch JM, Dalby CM, Robinson K, Stack C, Latimer PA, Hare JM, Olson EN, van Rooij E (2012). Inhibition of miR-15 protects against cardiac ischemic injury. Circ Res.

[16] Finnerty JR, Wang WX, Hebert SS, Wilfred BR, Mao G, Nelson PT (2010). The miR-15/107 group of microRNA genes: evolutionary biology, cellular functions, and roles in human diseases. J Mol Biol.

[17] Porrello ER, Mahmoud AI, Simpson E, Johnson BA, Grinsfelder D, Canseco D, Mammen PP, Rothermel BA, Olson EN, Sadek HA (2013). Regulation of neonatal and adult mammalian heart regeneration by the miR-15 family. Proceedings of the National Academy of Sciences of the United States of America.

[18] Xuan W, Liao Y, Chen B, Huang Q, Xu D, Liu Y, Bin J, Kitakaze M (2011). Detrimental effect of fractalkine on myocardial ischaemia and heart failure. Cardiovasc Res.

[19] Tijsen AJ, van der Made I, van den Hoogenhof MM, Wijnen WJ, van Deel ED, de Groot NE, Alekseev S, Fluiter K, Schroen B, Goumans MJ, van der Velden J, Duncker DJ, Pinto YM, Creemers EE (2014). The microRNA-15 family inhibits the TGFbeta-pathway in the heart. Cardiovasc Res.

[20] Zhu H, Yang Y, Wang Y, Li J, Schiller PW, Peng T (2011). MicroRNA-195 promotes palmitate-induced apoptosis in cardiomyocytes by down-regulating Sirt1. Cardiovasc Res.

[21] Katare R, Riu F, Mitchell K, Gubernator M, Campagnolo P, Cui Y, Fortunato O, Avolio E, Cesselli D, Beltrami AP, Angelini G, Emanueli C, Madeddu P (2011). Transplantation of human pericyte progenitor cells improves the repair of infarcted heart through activation of an angiogenic program involving micro-RNA-132. Circ Res.

[22] Shen L, Li J, Xu L, Ma J, Li H, Xiao X, Zhao J, Fang L (2012). miR-497 induces apoptosis of breast cancer cells by targeting Bcl-w. Experimental and therapeutic medicine.

[23] Guo ST, Jiang CC, Wang GP, Li YP, Wang CY, Guo XY, Yang RH, Feng Y, Wang FH, Tseng HY, Thorne RF, Jin L, Zhang XD (2013). MicroRNA-497 targets insulin-like growth factor 1 receptor and has a tumour suppressive role in human colorectal cancer. Oncogene.

[24] Yadav S, Pandey A, Shukla A, Talwelkar SS, Kumar A, Pant AB, Parmar D (2011). miR-497 and miR-302b regulate ethanol-induced neuronal cell death through BCL2 protein and cyclin D2. J Biol Chem.

[25] Roy S, Khanna S, Hussain SR, Biswas S, Azad A, Rink C, Gnyawali S, Shilo S, Nuovo GJ, Sen CK (2009). MicroRNA expression in response to murine myocardial infarction: miR-21 regulates fibroblast metalloprotease-2 via phosphatase and tensin homologue. Cardiovasc Res.

[26] Jeyaseelan K, Lim KY, Armugam A (2008). MicroRNA expression in the blood and brain of rats subjected to transient focal ischemia by middle cerebral artery occlusion. Stroke; a journal of cerebral circulation.

[27] van Rooij E, Sutherland LB, Liu N, Williams AH, McAnally J, Gerard RD, Richardson JA, Olson EN (2006). A signature pattern of stress-responsive microRNAs that can evoke cardiac hypertrophy and heart failure. Proceedings of the National Academy of Sciences of the United States of America.

[28] Abbate A, Bussani R, Amin MS, Vetrovec GW, Baldi A (2006). Acute myocardial infarction and heart failure: role of apoptosis. Int J Biochem Cell Biol.

[29] Voellenkle C, van Rooij J, Cappuzzello C, Greco S, Arcelli D, Di Vito L, Melillo G, Rigolini R, Costa E, Crea F, Capogrossi MC, Napolitano M, Martelli F (2010). MicroRNA signatures in peripheral blood mononuclear cells of chronic heart failure patients. Physiol Genomics.

[30] Zhu W, Zhu D, Lu S, Wang T, Wang J, Jiang B, Shu Y, Liu P (2012). miR-497 modulates multidrug resistance of human cancer cell lines by targeting BCL2. Med Oncol.

[31] Narula J, Haider N, Virmani R, DiSalvo TG, Kolodgie FD, Hajjar RJ, Schmidt U, Semigran MJ, Dec GW, Khaw BA (1996). Apoptosis in myocytes in end-stage heart failure. N Engl J Med.

[32] Liu L, Azhar G, Gao W, Zhang X, Wei JY (1998). Bcl-2 and Bax expression in adult rat hearts after coronary occlusion: age-associated differences. Am J Physiol Heart Circ Physiol.

[33] Chen Z, Chua CC, Ho YS, Hamdy RC, Chua BH (2001). Overexpression of Bcl-2 attenuates apoptosis and protects against myocardial I/R injury in transgenic mice. Am J Physiol Heart Circ Physiol.

[34] Peng X, Shao J, Shen Y, Zhou Y, Cao Q, Hu J, He W, Yu X, Liu X, Marian AJ, Hong K (2013). FAT10 protects cardiac myocytes against apoptosis. Journal of molecular and cellular cardiology.

[35] Cheng EH, Wei MC, Weiler S, Flavell RA, Mak TW, Lindsten T, Korsmeyer SJ (2001). BCL-2, BCL-X(L) sequester BH3 domain-only molecules preventing BAX- and BAK-mediated mitochondrial apoptosis. Molecular cell.

[36] Costa R, Morrison A, Wang J, Manithody C, Li J, Rezaie AR (2012). Activated protein C modulates cardiac metabolism and augments autophagy in the ischemic heart. J Thromb Haemost.

[37] Yan L, Vatner DE, Kim SJ, Ge H, Masurekar M, Massover WH, Yang G, Matsui Y, Sadoshima J, Vatner SF (2005). Autophagy in chronically ischemic myocardium. Proceedings of the National Academy of Sciences of the United States of America.

[38] Hamacher-Brady A, Brady NR, Gottlieb RA (2006). Enhancing macroautophagy protects against ischemia/reperfusion injury in cardiac myocytes. J Biol Chem.

[39] Matsui Y, Takagi H, Qu X, Abdellatif M, Sakoda H, Asano T, Levine B, Sadoshima J (2007). Distinct roles of autophagy in the heart during ischemia and reperfusion: roles of AMP-activated protein kinase and Beclin 1 in mediating autophagy. Circ Res.

[40] Luo T, Chen B, Zhao Z, He N, Zeng Z, Wu B, Fukushima Y, Dai M, Huang Q, Xu D, Bin J, Kitakaze M, Liao Y (2013). Histamine H2 receptor activation exacerbates myocardial ischemia/reperfusion injury by disturbing mitochondrial and endothelial function. Basic Res Cardiol.

[41] Biederbick A, Kern HF, Elsasser HP (1995). Monodansylcadaverine (MDC) is a specific *in vivo* marker for autophagic vacuoles. Eur J Cell Biol.

[42] Xu Q, Li X, Lu Y, Shen L, Zhang J, Cao S, Huang X, Bin J, Liao Y (2015). Pharmacologic modulation of autophagy to protect cardiomyocytes according to the time windows of ischemia/reperfusion. British journal of pharmacology.

