# Supplementary material for: Inhibition of microRNA-497 ameliorates anoxia/reoxygenation injury in cardiomyocytes by suppressing cell apoptosis and enhancing autophagy
Source: Oncotarget. 2015 Jul 29;6(22):18829–44. doi: 10.18632/oncotarget.4774 (PMC4643066; doi:10.18632/oncotarget.4774)
Supplement: Supplementary file 1 [file oncotarget-06-18829-s001.pdf]

## SUPPLEMENTARY FIGURES

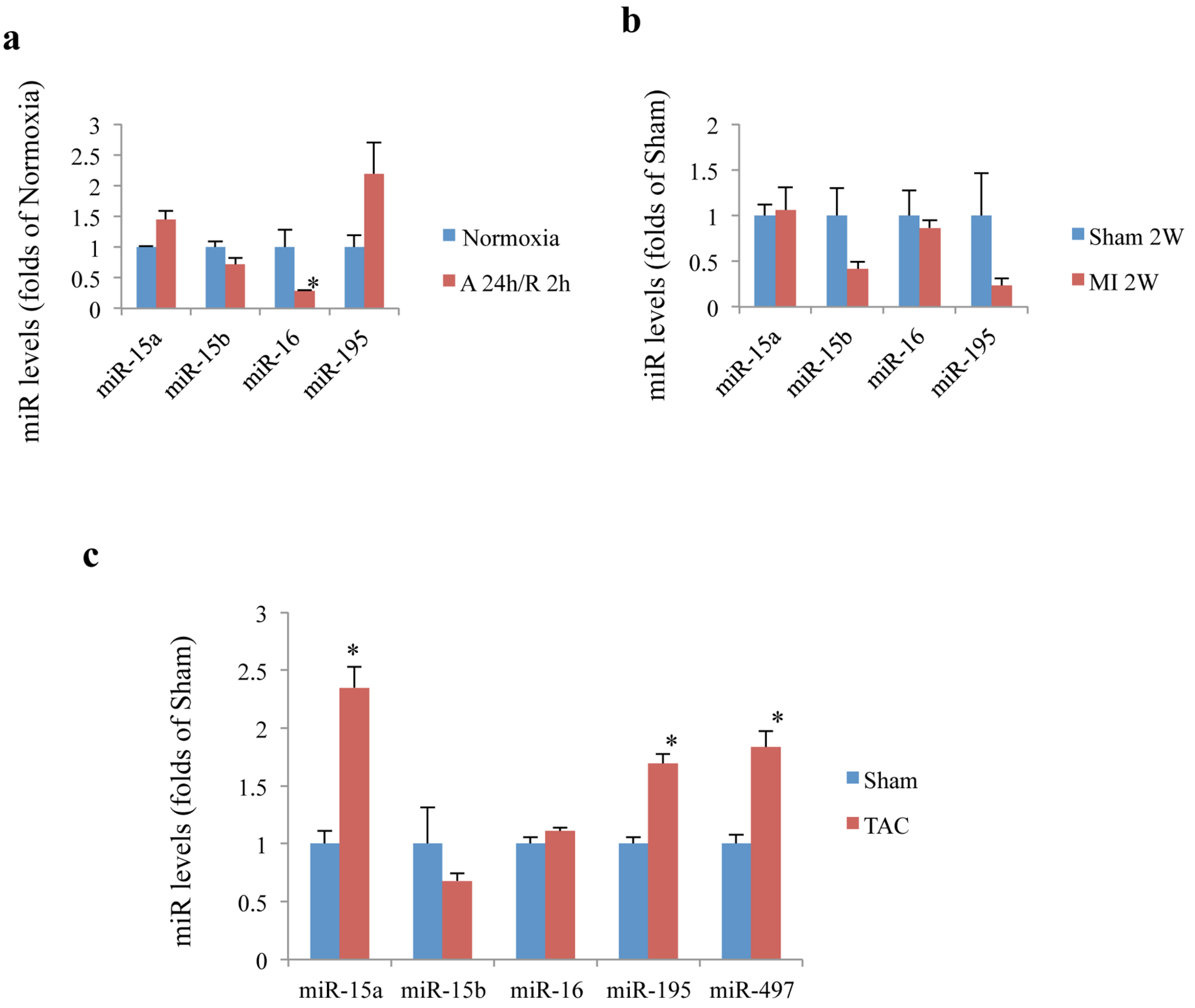


**Supplementary Figure S1: Real-time PCR-determined expression changes of miR-15 family members in response to different cardiac stresses. a.** In cultured neonatal rat cardiomyocytes exposed to anoxia/reoxygenation (AR). **b.** In mice subjected to myocardial infarction (MI) for 2 weeks. **c.** In mice subjected to transverse aortic constriction (TAC) for 4 weeks. $*P < 0.05$ vs. the corresponding normoxia or sham group, $n = 5–9$ in each group.

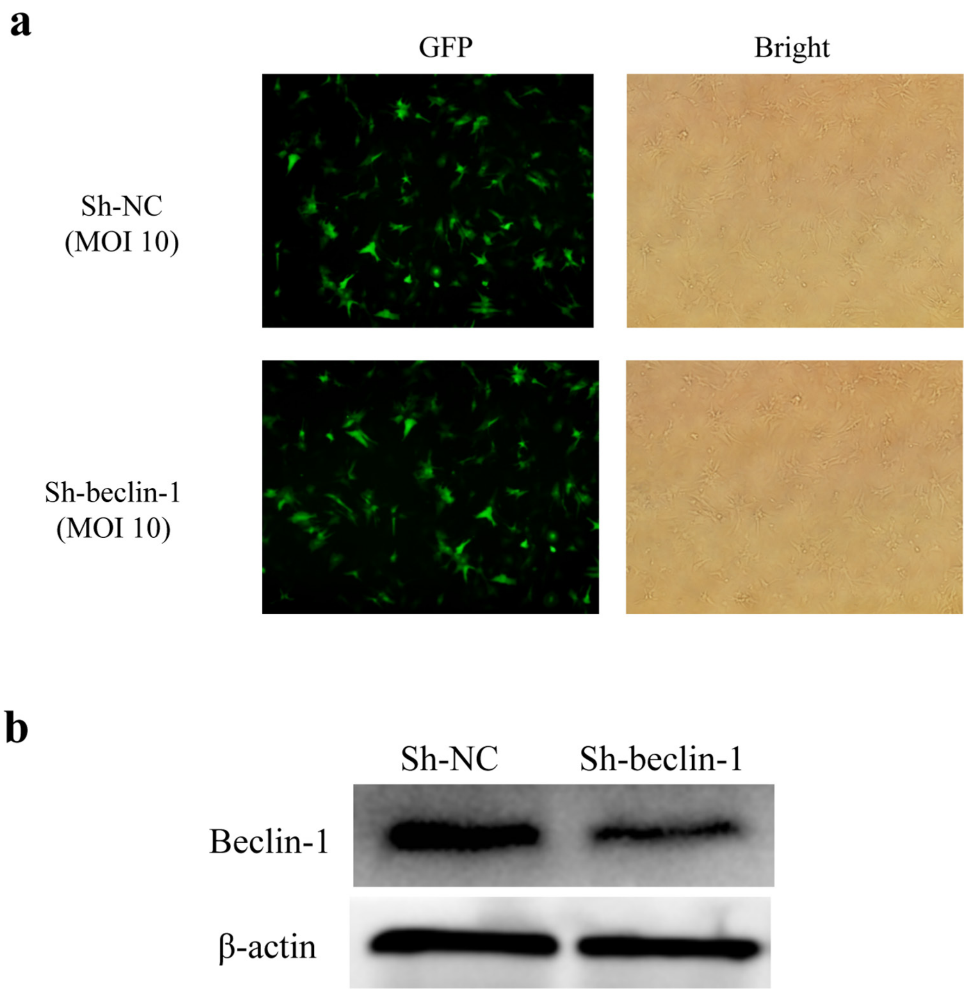


**Supplementary Figure S2: Infection efficiency of adenovirus carrying GFP, or sh-beclin-1 in cultured neonatal rat cardiomyocytes.** GFP, green fluorescent protein; MOI, multiplicity of infection; sh, short hairpin RNA; NC, negative control. **a.** about 90% of cardiomyocytes was infected by adenovirus as determined with fluorescent microscopy. **b.** Western blotting of beclin-1.
